# Artificial Intelligence (AI) for Early Diagnosis of Retinal Diseases

**DOI:** 10.3390/medicina60040527

**Published:** 2024-03-23

**Authors:** Uday Pratap Singh Parmar, Pier Luigi Surico, Rohan Bir Singh, Francesco Romano, Carlo Salati, Leopoldo Spadea, Mutali Musa, Caterina Gagliano, Tommaso Mori, Marco Zeppieri

**Affiliations:** 1 Department of Ophthalmology, Government Medical College and Hospital, Chandigarh 160030, India; upsparmar3112@gmail.com; 2 Massachusetts Eye and Ear, Department of Ophthalmology, Harvard Medical School, Boston, MA 02114, USA; 3 Department of Ophthalmology, Campus Bio-Medico University, 00128 Rome, Italy; 4 Fondazione Policlinico Universitario Campus Bio-Medico, 00128 Rome, Italy; 5 Department of Ophthalmology, University Hospital of Udine, p.le S. Maria della Misericordia 15, 33100 Udine, Italy; 6 Eye Clinic, Policlinico Umberto I, “Sapienza” University of Rome, 00142 Rome, Italy; 7 Department of Optometry, University of Benin, Benin City 300238, Edo State, Nigeria; 8 Faculty of Medicine and Surgery, University of Enna “Kore”, Piazza dell’Università, 94100 Enna, Italy; 9 Eye Clinic, Catania University, San Marco Hospital, Viale Carlo Azeglio Ciampi, 95121 Catania, Italy; 10 Department of Ophthalmology, University of California San Diego, La Jolla, CA 92122, USA

**Keywords:** artificial intelligence, AI, early diagnosis, retinal diseases

## Abstract

Artificial intelligence (AI) has emerged as a transformative tool in the field of ophthalmology, revolutionizing disease diagnosis and management. This paper provides a comprehensive overview of AI applications in various retinal diseases, highlighting its potential to enhance screening efficiency, facilitate early diagnosis, and improve patient outcomes. Herein, we elucidate the fundamental concepts of AI, including machine learning (ML) and deep learning (DL), and their application in ophthalmology, underscoring the significance of AI-driven solutions in addressing the complexity and variability of retinal diseases. Furthermore, we delve into the specific applications of AI in retinal diseases such as diabetic retinopathy (DR), age-related macular degeneration (AMD), Macular Neovascularization, retinopathy of prematurity (ROP), retinal vein occlusion (RVO), hypertensive retinopathy (HR), Retinitis Pigmentosa, Stargardt disease, best vitelliform macular dystrophy, and sickle cell retinopathy. We focus on the current landscape of AI technologies, including various AI models, their performance metrics, and clinical implications. Furthermore, we aim to address challenges and pitfalls associated with the integration of AI in clinical practice, including the “black box phenomenon”, biases in data representation, and limitations in comprehensive patient assessment. In conclusion, this review emphasizes the collaborative role of AI alongside healthcare professionals, advocating for a synergistic approach to healthcare delivery. It highlights the importance of leveraging AI to augment, rather than replace, human expertise, thereby maximizing its potential to revolutionize healthcare delivery, mitigate healthcare disparities, and improve patient outcomes in the evolving landscape of medicine.

## 1. Introduction

Artificial intelligence (AI) has been referred to as the fourth industrial revolution in mankind’s history [1]. The term was coined by John McCarthy in 1956, defining a branch of computer science dedicated to designing machines capable of learning and reasoning like humans to solve complex problems. Machine learning (ML), created by Arthur Samuel in 1959, is a field of AI where a program, when exposed to a vast amount of data, can learn to recognize specific patterns within that data [2]. This is achieved with the help of multiple interconnected algorithms layered together, each working on recognizing particular features. Collectively, this system is referred to as a neural network, as it attempts to simulate the functioning of neurons in the human brain.

Deep learning (DL) is a subdivision of machine learning wherein multiple artificial neural networks (ANNs) are layered together to better mimic the human brain’s processing capabilities [3]. Convolutional neural networks are a type of ANN that are widely used for image and video analysis [4]. The successful data interpretation by these programs can be reported in terms of sensitivity, specificity, or a receiver operating characteristic (ROC) curve, which plots the true positive rate against the false positive rate [5].

The past two decades have witnessed a surge in AI-powered solutions within the medical field. Digital images and numerical data are frequently used to train AI and ML algorithms. The unique nature of the retina, being readily accessible through various imaging techniques like fundus photography and optical coherence tomography (OCT), has positioned it as an ideal candidate for AI-assisted diagnosis. With 30 million OCT scans performed annually in the US alone, this field of ophthalmology provides a vast repository of raw data for algorithm training [6].

The significant variability and progressive nature of retinal diseases typically necessitate multiple consultations for an accurate diagnosis, constant monitoring, and personalized management, thereby straining both time and resources [7]. This situation can lead to delayed diagnosis and suboptimal visual outcomes. AI seeks to efficiently analyze the large amount of patient data generated, aiming to reduce patient burden, facilitate early diagnosis, enhance management strategies, and ultimately improve long-term prognosis.

## 2. Materials and Methods

The review was conducted utilizing PubMed (https://pubmed.ncbi.nlm.nih.gov) and Reference Citation Analysis (RCA) (https://www.referencecitationanalysis.com). PubMed, a widely used and trusted biomedical literature database maintained by the National Library of Medicine (NLM), was chosen as the primary database for this research endeavor. Its extensive coverage of peer-reviewed journals in the field of medicine and life sciences makes it an ideal resource for retrieving relevant scientific literature. Research was conducted using a combination of search terms. These terms included variations of “Artificial Intelligence”, “Machine Learning”, and “Deep Learning”, combined with terms related to ophthalmology and specific retinal diseases, such as diabetic retinopathy (DR), age-related macular degeneration (AMD), Macular Neovascularization, retinopathy of prematurity (ROP), retinal vein occlusion (RVO), hypertensive retinopathy (HR), Retinitis Pigmentosa, Stargardt disease, best vitelliform macular dystrophy, and sickle cell retinopathy. Boolean operators (AND, OR, NOT) were utilized to combine these terms logically, ensuring comprehensive coverage of the relevant literature while minimizing irrelevant results. The search was limited to articles written in English to ensure relevance and accessibility. Titles and abstracts of retrieved articles were manually screened to select those relevant to the study objectives. Full texts of selected articles were then reviewed to extract information on AI models utilized, performance metrics reported, clinical implications discussed, and challenges associated with AI integration in clinical practice. Additionally, manual searches of reference lists from relevant articles were conducted to supplement electronic database searches, and citation tracking was utilized to identify additional relevant studies citing key articles. The comprehensive search strategy employed in this study aimed to ensure the inclusion of all of the relevant literature on the topic, thus providing a thorough understanding of AI applications in retinal disease diagnosis and management.

## 3. Applications of Artificial Intelligence in Retina

### 3.1. Diabetic Retinopathy

Diabetic retinopathy (DR), a microvascular complication of Diabetes Mellitus, stands as a leading cause of preventable blindness in the working-age population, with an estimated prevalence of 28.54 million facing vision-threatening complications [8]. The American Academy of Ophthalmology (AAO) Preferred Practice Patterns recommends annual screening for DR [9]. AI-based DR screening systems aim to reduce costs and improve patient access to screening. These systems utilize algorithms to detect early signs of DR from color fundus photography. These features include microaneurysms [10], red lesions [11,12], hemorrhages, and blood vessel segmentation [13,14].

The IDx-DR (Idx, Iowa City, IA, USA) was the first AI system approved by the Food and Drug Administration for the detection of DR [15]. Using color fundus photos, this system effectively identifies specific biomarkers of DR, providing guidance on whether a visit to an ophthalmologist is necessary or if a follow-up screening next year is sufficient [16]. In the published literature, the system has demonstrated impressive results across diverse populations, including North African, Caucasian, and Sub-Saharan groups [17]. Using the DL-enhanced algorithm with IDx-DR against the publicly available Messidor-2 dataset, the sensitivity and specificity of the system were found to be 96.8% and 87% [18]. In its pre-registered clinical trial, the IDx-DR reported a sensitivity and specificity of 87.2% and 90.9% in detecting more than mild DR [16].

In Europe, the EyeArt (EyeNuk Inc., Woodland Hills, CA, USA) and Retmarker (Retmarker Ltd., Voimbra, Portugal) systems are approved as AI-based class IIa medical devices to assist in DR screening [19,20]. EyeArt analyzes retinal images to determine the necessity of referral for diabetic retinopathy. The system is reported to have a sensitivity and specificity of 91.7% and 91.5% [20]. Interestingly, in a study by Rajalakshmi R et al., the software was tested with smartphone-based fundus images, showing sensitivities and specificities of 99.3% and 68.8% for referable DR and 99.1% and 80.4% for sight-threatening DR, respectively [20]. The Retmarker software, developed in Portugal, is unique in its ability to compare fundus photographs from the current screening to previous screenings and comments on disease progression [21,22]. In a study using data from over twenty thousand patients, the software was found to have a sensitivity of 85% and 97.9% for referable and proliferative DR [23]. Additionally, the software tracks the rate of new microaneurysm formation, which can signal worsening diabetic retinopathy [21]. Comparative analysis revealed that EyeArt achieved a higher sensitivity compared to Retmarker when analyzing the same dataset (93.8% as compared to 85%). The reported false-positive rate, however, was also significantly higher for EyeArt (80.1% compared to 53.3%) [24].

Islam et al. developed an AI model based on the principle of supervised contrastive learning to detect DR from fundus photographs. The model was validated against the APTOS 2019 blindness detection dataset and achieved an AUC of 0.9850 and an accuracy of 98.36% [25]. Another study proposed an AI model to help diagnose DR using OCT scans. They used 188 scans to help validate their algorithm and reported an accuracy of 96.81% for DR diagnosis [26]. Gulshan et al. were sponsored by Google to train a CNN-based AI model to help detect referable DR. Their model was tested using the EyePACS-1 and Messidor-2 dataset and was reported to achieve an AUC of 0.991 and 0.990, respectively [27]. Zhang et al. in 2022 developed a CNN-based system that would automatically classify DR using fundus images. They trained and validated this model against the Messidor-2 and EyePACS-1 and reported an accuracy, sensitivity, and specificity of 89.9%, 88.2%, and 91.3% with the EyePACS-1 dataset and 91.8%, 90.2%, and 93% with the Messidor-2 dataset [28]. A summary of the various studies that have developed AI models to aid with the diagnosis of DR has been tabulated in Table 1.

### 3.2. Age-Related Macular Degeneration

Age-related macular degeneration (AMD) is the leading cause of irreversible vision loss in the elderly population in developed countries. Affecting nearly 9% of individuals between ages 45 and 85 globally, the prevalence of AMD is projected to reach a staggering 288 million by 2040 [35,36]. Notably, up to 84% of cases remain undiagnosed in the early stages, often due to the absence of symptoms [37]. To address this, the AAO recommends regular biennials for individuals aged 65 and above, underscoring the growing need for AI-driven solutions to support large-scale screening efforts and alleviate the burden on clinicians [38].

In 2013, Grinsven et al. introduced an ML system designed to detect and quantify drusen from color fundus photographs. Based on the detected drusen, the algorithm assessed the risk of developing advanced AMD [39]. It achieved an ROC curve of 0.948 and had a similar performance when compared to ophthalmologists in detecting drusen. Burlina et al. developed deep CNNs to analyze fundus photographs for automated AMD severity grading. The accuracy values with which the system was able to classify different classes of AMD were reported to be 79.4%, 81.5%, and 93.4% [40]. Chou et al. validated their DL model against 699 fundus photographs to diagnose AMD and reported an accuracy, sensitivity, and specificity of 83.67%, 80.76%, and 84.72%, respectively. In addition to disease diagnosis, AI models have also been used to predict disease severity and progression in patients with AMD [41]. Waldstein SM et al. used AI algorithms to analyze drusen volumes and hyperreflective foci volumes in the OCT scans as biomarkers for AMD progression [42]. Yan et al. used a CNN to develop an AI model to help predict disease progression in AMD patients. They tested their model against 31,262 OCT images and reported an AUC of 0.85 [43,44].

AI-driven methodologies have also been used to distinguish geographic atrophy from conditions that mimic AMD, such as extensive macular atrophy with pseudodrusen-like appearance (EMAP), a severe and rapidly progressive form of macular degeneration predominantly affecting middle-aged individuals [45,46,47]. Specifically, Chouraqui M et al. utilized a DL classifier based on the ResNet-101 design, pre-trained with 30° × 30° and 55° × 55° FAF images, to differentiate these two conditions [48]. The authors trained the network with images from 135 EMAP and 185 AMD patients and achieved good-to-excellent results, particularly using the 55° × 55° classifier (sensitivity 90%, specificity 84.6%) (Table 2).

### 3.3. Macular Neovascularization, Diabetic Macular Edema, and Other Macular Diseases

Optical coherence tomography (OCT) scans serve as the cornerstone to diagnose and monitor a wide range of macular disorders. Machine learning algorithms excel at analyzing images with intricate details and consistent views. These features make OCT scans particularly amenable for this application due to their high level of detail and uniformity within the captured area. Some of the widely studied biomarkers include subretinal fluid (SRF), intraretinal fluid (SRF), and pigment epithelial detachment (PED) [53].

Schlegl et al. developed an algorithm capable of identifying retinal fluid on OCT scans, further distinguishing between subretinal and intraretinal fluid. When tested against 1200 OCT scans from patients with neovascular age-related macular degeneration (nAMD) and diabetic macular edema, the model achieved an AUC of 0.98 for detecting subretinal fluid and 0.94 for detecting intraretinal fluid [44]. Han et al. developed an AI model using a CNN to diagnose nAMD and also compared the diagnostic accuracy of the model to that of ophthalmologists. The study cohort included 4749 spectral domain optical coherence tomography images and the model achieved an accuracy of 87.4%, which was at par with the ophthalmologists [54]. Song et al. used a CNN to develop an AI model to predict nAMD. They trained and tested the algorithm against 671 spectral domain optical coherence tomography images and reported an accuracy, sensitivity, and specificity of 93%, 87.3%, and 92.2%, respectively [55]. Romo-Bucheli D et al. developed a DL model using DenseNet and a recurrent neural network (RNN) that analyzed OCT scans to predict treatment requirements in patients with nAMD. The model achieved an AUC of 0.85 and 0.81 in detecting patients with low and high treatment requirements [56] (Table 3).

### 3.4. Retinopathy of Prematurity

Retinopathy of prematurity (ROP) is among the leading causes of childhood blindness worldwide. It is characterized by abnormal vascular proliferation, predominantly affecting premature infants, with an incidence rate reported to be as high as 68% in the premature population [63,64]. The International Classification of ROP (ICROP) currently classifies ROP as a “Plus” disease, severe ROP that requires prompt treatment, and a “Pre-plus” disease, a less severe form of the disease. This classification is based on biomarkers, such as arterial tortuosity and venous dilation, for accurate disease assessment [65].

Wang et al. developed DeepROP, a DNN-based system employing two CNN classifiers (Id-Net and Gr-Net) to facilitate early detection of ROP using retinal fundus photographs. Id-Net is designed to identify features and cases of ROP, whereas Gr-Net categorizes the severity of ROP in these cases as minor or severe. Remarkably, both of these classifiers achieved high sensitivity (99.62% for Id-Net and 88.46% for Gr-net) and specificity (99.32% for Id-net and 92.31% for Gr-net) [61]. Wu Q et al. also developed and validated a DL algorithm for the detection (OC-Net) and grading (SE-net) of ROP. The mean AUC was reported to be 0.90 and 0.87 for OC-Net and SE-net, respectively [60]. Redd KT et al. tested the i-ROP DL severity score in their cohort of 870 infants. They reported an AUC of 0.960 along with a sensitivity and specificity of 94% and 79%, respectively [66] (Table 3).

### 3.5. Retinal Vein Occlusion

Retinal vein occlusion (RVO) ranks as the second most common retinal vascular disease after diabetic retinopathy [67,68]. It is characterized by sudden painless loss of vision. The disease is further classified into central, branch, and hemicentral retinal vein occlusion, with the branch of RVO being the most common variant [59]. Diagnostic features observable on fundus photographs include exudates, microaneurysms, superficial and deep hemorrhages, telangiectatic vessels, and sclerosed veins [59,69,70]. Early and accurate diagnosis of RVO is crucial, as it may enable timely intervention and potentially prevent severe visual impairment in these patients.

Chen J. S et al. developed a DL system using four different AI algorithms for the early screening of RVO [71]. These algorithms were tested on a cohort of 8600 color fundus photographs, and their Inception-v3 model was reported to have a sensitivity and specificity of 99% and 95%, respectively. Abitbol et al. tested DenseNet121, an AI-based system to diagnose retinal vascular disorders, including RVO. The model reported an AUC of 0.912 and an accuracy of 88.4% while analyzing a study cohort of 224 ultra-widefield color fundus images [72]. Nagasato et al. validated two AI models using 465 ultrawide-field fundus photographs and reported a sensitivity and specificity of 94% and 97%, respectively [73]. Kang et al. worked on a study cohort of 2992 eyes using a CNN-based AI model and achieved an accuracy, sensitivity, and specificity of 97.7%, 96%, and 98% for the diagnosis of RVO [74] (Table 3).

### 3.6. Hypertensive Retinopathy

Uncontrolled high blood pressure, or systemic hypertension, can damage various organs, including the retina. Persistent high pressure can strain the endothelium of retinal blood vessels, induce compensatory smooth muscle cell hypertrophy in the arterial walls, and eventually cause the narrowing of vessel lumens [75,76]. The long-term sequelae of this process, observable through fundus examination, include intraretinal exudates, cotton wool spots, and flame-shaped hemorrhages [77].

In the recent literature, many AI-based systems have been tested to effectively screen and grade patients with hypertensive retinopathy (HR). In 2022, Dong et al. used CNNs to develop an algorithm to diagnose various retinal pathologies, including HR. The algorithm was validated using more than 120,000 fundus photographs and was reported to achieve an AUC of 0.837 for the diagnosis of HR [78]. Han et al. used DL to develop an anomaly detection model to screen for ocular pathologies. The model was tested on ninety-thousand fundus photographs and achieved an AUC of 0.895, with a sensitivity and specificity of 81.2% and 82.7% for the diagnosis of HR [57]. Akbar et al. used DL to develop an AI model to aid with grading HR in patients. The model was validated against three different patient datasets, AVRDB, VICAVR, and INSPIRE-AVR, and was found to achieve an accuracy of 98.1%, 95.6%, and 95.1% with each of them, respectively [79]. Abbas et al. in 2021 used DenseNet to create HYPER-RETINO to help classify HR. Their algorithm was tested on 1400 fundus photographs and was reported to have a sensitivity and specificity of 90.5% and 91.5% [80] (Table 3).

### 3.7. Retinitis Pigmentosa

Retinitis Pigmentosa (RP), or rod–cone dystrophy, is the most common inherited retinal disorder, characterized by the progressive atrophy of rod photoreceptors followed by secondary degeneration of cones [81]. The current global incidence of RP is reported to be between 1 in 2000 and 1 in 4000 individuals [82]. Clinically, the disease presents with impaired night vision and narrowing of the visual field. As the disease progresses, patients experience progressive vision loss and decreased contrast perception. The clinical triad of bone spicules in the retinal periphery, a pale optic disk, and vessel narrowing on fundoscopy represent the current mainstay of diagnosis and the gateway to genetic testing [82]. Some of the recent studies have used AI to aid with this image processing for an early and accurate diagnosis of the disease.

Chen T et al. developed a DL model to accurately detect RP using color fundus photographs of patients. Their model was reported to achieve an accuracy of 96.00%, which was comparable with that of ophthalmologists when they examined the same images [83]. Nagasato D et al. studied five DL models, Visual Geometry Group-16, Residual Network-50, Inception V3, DenseNet121, and EfficientNetB0, to estimate visual function in patients with RP. These models were validated against ultra-widefield fundus autofluorescence images from 695 patients and were found to accurately estimate the visual acuity and central sensitivity in these patients (*p* < 0.001) [84]. Liu TYA et al. trained their DL algorithm to predict visual impairment in patients with RP. They tested their model against two different patient datasets and achieved an AUC of 0.83 and 0.78, respectively [85]. Arsalan et al. developed a DL-based segmentation network (RP segmentation network; RPS-Net) to accurately detect pigment in color images and was reported to have an accuracy of 99.5% [86] (Table 3).

### 3.8. Stargardt Disease

Stargardt disease, the most common monogenic retinal dystrophy, affects approximately 1 in 6578 individuals [87,88]. The disease is primarily caused by biallelic mutations in the ABCA4 gene, leading to the abnormal accumulation of bisretinoids in the RPE and subsequent degeneration of photoreceptors and RPE cells [89]. The large genetic variability of this complex gene contributes to the wide range of phenotypic heterogeneity observed in Stargardt disease [90]. Nonetheless, typical funduscopic alterations characteristic of the disease include (1) macular atrophy, (2) flecks, and (3) peripapillary sparing [88].

Recent AI approaches applied to Stargardt disease primarily leverage fundus autofluorescence (FAF) and OCT scans. Wang et al. pioneered the use of a deep learning CNN system (U-Net) for the semantic segmentation of Stargardt atrophic lesions using FAF images [91]. Initially trained to distinguish Stargardt FAF images from those of healthy controls, the system was subsequently expanded to include FAF images from patients with AMD. This approach yielded promising segmentation outcomes compared to manual grading, achieving a DICE similarity coefficient and an overlapping ratio of 0.87 + 0.13 and 0.78 + 0.17, respectively.

For OCT imaging, retinal layer segmentation has predominantly employed graph-based methods to detect atrophic areas and flecks associated with Stargardt disease. Utilizing a supervised AI deep learning framework, Mishra et al. implemented a 12-retinal layer algorithm that demonstrated subpixel accuracy in analyzing OCT scans from the ProgSTAR study, showcasing the potential of AI in enhancing diagnostic precision and understanding of Stargardt disease [92].

### 3.9. Best Vitelliform Macular Dystrophy

Best vitelliform macular dystrophy (BVMD) is the second most common macular dystrophy with an estimated prevalence of 1 in 10,000 in the United States. The disease is characterized by dominantly inherited mutations in the BEST1 gene on chromosome 11, leading to the production of a dysfunctional bestrophin protein. Malfunctioning of this chloride channel, situated on the basolateral membrane of RPE cells, results in the subretinal accumulation of lipofuscin and unphagocytosed photoreceptor outer segments [93]. Clinically, this condition is marked by the characteristic “egg yolk” (vitelliform) macular lesion and a reduced Arden ratio (<1.5) on electrooculogram [94]. Although the original classification relied on fundus examination and photography, recent advances in retinal imaging have deepened our understanding of BVMD pathophysiology, leading to more accurate diagnosis and the development of novel staging systems [95,96,97,98].

In the realm of AI approaches, efforts to distinguish BVMD from Adult Vitelliform Lesions (AVLs) have shown promising results. Crincoli et al. classified FAF and OCT images from 182 BVMD eyes and 96 eyes with AVLs using the Inception-ResNet-v2 CNN [99]. This study achieved a 90% accuracy in differentiating the two conditions using deep learning classifiers on both ImageJ-processed and unprocessed images, surpassing human diagnostic performance.

### 3.10. Sickle Cell Retinopathy

Sickle cell disease (SCD), caused by a mutation in the β-globin gene of hemoglobin, is one of the most common inherited blood disorders [100]. This mutation causes the erythrocytes to change from their disc shape to a sickle shape during periods of ischemia, resulting in microvascular occlusions in the body [101]. SCD patients face a risk of vision loss due to blocked blood vessels in the retina [102]. Chronic tissue ischemia from these microvascular blockages can precipitate severe ocular complications, including abnormal new blood vessel growth (neovascularization), vitreous hemorrhage, and detachment of the retina [103]. Considering these risks, early detection and preventive measures are crucial to avoid vision loss in these patients. Therefore, dilated fundus examinations, which allow detailed examination of the retina, are currently recommended for SCD patients starting at age 10 [104].

Innovations in AI have shown promise in enhancing the diagnostic accuracy for SCD-related ocular conditions. Cai S et al. trained a CNN to detect the classic sea fan neovascularization in patients with SCD using ultra-widefield color fundus photographs. In their study, the CNN was able to achieve an AUC of 0.988 with a sensitivity and specificity of 97.4% and 97%, respectively [105]. Sevgi DD et al. used a DL algorithm to analyze vascular and ischemic parameters, like the ischemic index, vessel length, and area in patients with SCD. The imaging modality used was ultra-widefield fluorescein angiography scans. They concluded that the DL algorithm was more accurate at detecting these parameters compared to alternative image processing systems [106]. Alam M et al. used three different AI algorithms to evaluate sickle cell retinopathy biomarkers in optical coherence tomography angiography (OCTA) scans. All three algorithms achieved high sensitivity specificity and accuracy while testing (Table 1) [62].

## 4. Challenges and Pitfalls to the Use of AI

One of the major challenges in using AI for disease diagnosis is the “black box phenomenon”. This term describes AI systems whose internal mechanisms are opaque, meaning their internal workings are difficult or impossible to understand. Users are left with the inputs (data fed into the system) and outputs (the resulting diagnosis or prediction), but the reasoning process behind these outputs remains hidden [107,108]. The accessibility and affordability of these systems can also pose a challenge to the developing world, even though the main concern regards the image quality and lack of complete datasets, in addition to false positive and false negative outcomes. Developing, validating, testing, and implementing these AI models require substantial financial resources, which can be a major limiting factor [24].

Assessing the cost impact of AI in retinal disease diagnosis remains challenging, given its limited routine use. However, preliminary evidence suggests that AI can reduce costs by improving diagnostic accuracy and streamlining workflows [23]. However, further comprehensive analysis should explore these potential savings alongside the clinical impact of AI integration, considering factors such as workflow efficiency, diagnostic accuracy, and patient outcomes. By understanding both the economic and clinical implications, stakeholders can better leverage AI technologies to enhance patient care and optimize resource utilization.

Beyond the “black box phenomenon”, where the inner workings of AI systems remain opaque, concerns extend to image quality and misclassifications, which significantly impact diagnostic accuracy. For instance, misidentifying vessels or swapping arteries and veins can lead to erroneous conclusions. Additionally, while AI systems offer valuable insights, the responsibility for final decision making still rests with clinicians, highlighting the need for transparency and accountability in their use.

Another aspect is the fact that diagnostic assessments for retinal diseases are now paid for and supported by several private healthcare insurers. Therefore, there are implications for the patient not only from identifying issues but also increased fees due to potentially higher risk scores.

A critical concern with AI models is their potential to perpetuate existing social and racial biases if trained exclusively on data from specific demographic groups. Therefore, as expected, it is important to incorporate data from various ethnicities and nationalities to increase the generalizability of these systems in real-world scenarios [109]. We need ethnicity or other population-based characteristic feeds, as this is important for risk profiling and patient management. It is also important to note that while these systems excel at analyzing specific data points, they can overlook crucial aspects of a patient’s health that a comprehensive patient–physician interaction uncovers. Additionally, the lack of a physical examination and focus on specific disease biomarkers can cause these systems to overlook the broader picture [110].

## 5. Future Perspectives

In addition to examining current applications and challenges, this review also endeavors to outline future perspectives and research directions in the field of AI for retinal disease diagnosis. Looking ahead, there is a burgeoning interest in exploring novel AI techniques, such as federated learning and transfer learning, to enhance the performance and generalizability of AI models across diverse populations and clinical settings. Furthermore, integrating multimodal imaging data, including OCT, fundus photography, and angiography, holds promise for improving diagnostic accuracy and expanding the scope of AI-driven retinal disease assessment. Additionally, the development of interpretable AI models that provide transparent decision-making processes is crucial for enhancing trust and acceptance among clinicians and patients. Moreover, there is a growing emphasis on leveraging AI not only for diagnosis but also for personalized treatment planning and monitoring of retinal diseases, ushering in a new era of precision medicine in ophthalmology. Collaborative efforts between clinicians, researchers, and industry partners are essential for advancing AI technologies and translating them into clinically impactful tools that ultimately benefit patients worldwide. By addressing these future perspectives and research directions, this review aims to catalyze continued innovation and progress in the field of AI-driven retinal disease diagnosis.

## 6. Conclusions

In the face of expanding patient populations and limited healthcare resources, the integration of artificial intelligence (AI) into retinal disease diagnosis offers a promising solution. AI algorithms, adept at analyzing complex retinal imaging data, enhance diagnostic accuracy and efficiency. However, it is crucial to recognize that AI should complement rather than replace human expertise. The collaborative synergy between AI and clinicians optimizes diagnostic precision, leading to improved patient outcomes. This collaborative paradigm holds the potential to revolutionize retinal disease diagnosis, mitigating healthcare disparities and advancing towards a healthier future for all.

## Figures and Tables

**Table 1:** Summary of selected studies using artificial intelligence in the diagnosis, stages, and prognosis of diabetic retinopathy (DR).

Study	Disease	AI Tool	Study Cohort/Database	Imaging Analyzed	Performance Metrics
Pires et al. [29]	DR diagnosis	CNN	Messidor-2 (1748 images), Kaggle (88,702 images), DR2 (520 images)	CFP	Accuracy = 98.2% (Messidor-2), 98% (DR2)
Jiang et al. [30]	DR diagnosis	CNN	30,244 images	CFP	AUC = 0.946 Sensitivity = 85.57% Specificity = 90.85% Accuracy = 88.21%
Esfahani et al. [31]	DR diagnosis	ResNet-34 (CNN)	Kraggle (35,000 images)	CFP	Sensitivity = 85% Specificity = 86%
Abramoff et al. [18]	DR staging	CNN	Messidor-2 (1748 images)	CFP	AUC = 0.98 Sensitivity = 96.8% Specificity = 87%
Pratt et al. [32]	DR staging	CNN	Kraggle (35,000 images)	CFP	Sensitivity = 30% Specificity = 95% Accuracy = 75%
Zhang F et al. [33]	DR grading	ResNet-34, Inception v3 (CNN)	1089 images	CFP	AUC = 0.958 Kappa = 0.860
Katz et al. [34]	DR grading	W-net (CNN)	6981 images	CFP	Accuracy 98.9%

DR—diabetic retinopathy, CFP—color fundus photographs, CNN—convolutional neural network, AUC—area under the curve.

**Table 2:** Summary of selected studies using artificial intelligence in the diagnosis, stages, and prognosis of age-related macular degeneration (AMD).

Study	Disease	AI Tool	Study Cohort/Database	Imaging Analyzed	Performance Metrics
Burlina et al. [40]	AMD diagnosis	CNN	67,401 images	CFP	AUC = 0.970 Sensitivity = 83.1% Specificity = 93.6% Accuracy = 90.2%
Bhuiyan et al. [49]	AMD progression	DL	>4600 participants from AREDS	CFP	Sensitivity 91% (year 1), 92% (year 2) Specificity = 85% (year 1), 84% (year 2) Accuracy = 86% (year 1), 85% (year 2)
Banerjee et al. [50]	AMD progression	DL	13,954 images	OCT	AUC = 0.96
Schmidt-Erfurth et al. [51]	AMD progression	DL	495 images	OCT	AUC = 0.68
Lee et al. [52]	AMD diagnosis	DNN	48,312 cases, 52,690 controls	OCT	AUC = 0.98 Sensitivity = 84.6% Specificity = 91.5% Accuracy = 87.6%

CFP—color fundus photographs, AMD—age-related macular degeneration, CNN—convolutional neural network, DL—deep learning, DNN—deep neural network, OCT—optic coherence tomography, AUC—area under the curve.

**Table 3:** Summary of selected studies using artificial intelligence in the diagnosis, stages, and prognosis of the other retinal diseases included in this study.

Study	Disease	AI Tool	Study Cohort/Database	Imaging Analyzed	Performance Metrics
Han et al. [57]	HR screening	Anomaly detection (AD) model (DL)	90,499 images	CFP	AUC = 0.895 Sensitivity = 81.29% Specificity = 82.75% Accuracy = 82.37%
Arsalan et al. [58]	HR	CNN	DRIVE, CHASE-DB1, STARE (1960 images)	CFP	AUC = 0.9697 Sensitivity = 85.26% Specificity = 97.91% Accuracy = 98.83%
Chen Q [59]	RVO	ResNet-50 (CNN)	600 CFPs from 481 pateints	CFP	AUC 1 Sensitivity—100% Specificity—89%
		Inception-v3 (CNN)			AUC 0.99 Sensitivity—100% Specificity—97%
		DenseNet-121 (CNN)			AUC 1 Sensitivity—99% Specificity—92%
		SE-ReNeXt-50 (CNN)			AUC 1 Sensitivity—100% Specificity—91%
Wu Q [60]	ROP	OC-Net (DL)		CFP	AUC—0.90 Accuracy—52.8% Sensitivity—100% Specificity—37.8%
		SE-Net (DL)			
Wang J [61]	ROP	Id-Net (DeepROP) (DNN)		CFP	Sensitivity—96.62% Specificity—99.32%
		Gr-Net (DeepROP) (DNN)			Sensitivity—88.46% Specificity—92.31%
Alam M [62]	SCR	Support vector machine		OCTA	Sensitivity—100% Specificity—100% Accuracy—100%
		K-nearest neighbor		OCTA	Sensitivity—95% Specificity—93% Accuracy—93%
		Discriminant analysis		OCTA	Sensitivity—93% Specificity—92% Accuracy—92%

CFP—color fundus photographs, RVO—retinal vein occlusion, DL—deep learning, CNN—convolutional neural learning, ROP—retinopathy of prematurity, DNN—deep neural network, SCR—sickle cell retinopathy, OCTA—optic coherence tomography angiography, AUC—area under the curve.
